# Preservative effects of some selected spice oleoresins to stabilize the sunflower oil in comparison to tertiary butylhydroquinone

**DOI:** 10.1002/fsn3.555

**Published:** 2017-12-01

**Authors:** Mahalaxmi Pradhananga, Pratibha Manandhar

**Affiliations:** ^1^ Central Campus of Technology Tribhuvan University Dharan Nepal; ^2^ Sunsari Technical College Tribhuvan University Dharan Nepal

**Keywords:** antioxidant, oleoresins, oxidation and rancidity

## Abstract

DPPH (1, 1‐Diphenyl‐2‐Picrylhydrazyl) radical scavenging activity was performed to find out the antioxidant activity (AA) of all the antioxidants used at a concentration of 5, 10, and 15 μl/ml. Effect of spice oleoresins (clove, black pepper, and ginger) (200 ppm) and TBHQ (200 ppm) were incorporated in stabilizing refined, bleached, and deodorized (RBD) sunflower oil heating at frying temperature (170°C) and during accelerated oxidation storage (70°C) was studied in comparison to Tert‐Butylhydroquinone (TBHQ). Antioxidant activity (AA) was found to be in the order TBHQ > clove > ginger > pepper at a concentration of 5; 10; 15 μl/ml. A direct correlation was found between AA and the effectiveness of oleoresins and TBHQ in controlling AV and PV of the sunflower oil. During both heating and storage, TBHQ was found most effective to control AV and PV followed by clove, pepper, and ginger.

## 1 INTRODUCTION

Rancidity, simply can be said to be the subjective organoleptic appraisal of the off flavor quality of food. Rancid off flavors are concerned with the changes that result from reaction of fats/oils with atmospheric oxygen, that is, oxidative rancidity or by hydrolytic reactions catalyzed by moisture from food or from microorganisms (Lundberg, 1961). Frying is one of the most common cooking techniques used in domestic and industrial food preparation. Flavor, shelf‐life, and nutrient composition of fried food and vegetable oils are altered by this process, and also some of the compounds formed may have undesirable consequences on consumers’ health (Hosseini, Ghorbani, Meshginfar, & Mahoonak, 2016). Unfortunately, fats and oils are oxidative in nature even at room temperature (Potter, 1987).

Autoxidation is the major cause of rancidity of fats and oils (Allen & Hamilton, 1983).Oxidation not only decreases the stability of oils and fats during long‐term storage but also negatively affects the stability of lipid containing foods, in particular those which were thermally processed like fried foodstuffs (Anwar, Jamil, Iqbal, & Sheikh, 2006). Synthetic antioxidants such as tertbutylhydroquinone (TBHQ) are often used to retard lipid oxidation in food systems. Due to their low thermal stability, the concern about their long‐term effects on human health and the increasing demand by consumers for natural products, plant extracts emerged as good alternatives to synthetic antioxidants (Redondo‐Cuevas, Castellano, & Raikos, 2017). Among natural antioxidants, the spices and herbal extracts, tocopherols, ascorbic acid, citric acid, and carotenoids are widely covered. It is commercially available in the market in different formulations such as oleoresin, seasoning, and flavoring agent (Upadhyay & Mishra, 2016). Natural antioxidants are effective in thermo‐oxidative stabilization of fats and oils (Jaswir, Che Man, & Kitts, 2000).

In this study, we aimed to identify the natural antioxidant capacity among the used spices (black pepper, ginger, and clove) suitable for increasing the oxidative stability of commercially available sunflower oil commonly used for food applications. The effectiveness of the most potent spices as an antioxidant was evaluated by assessing the heating performance and accelerated oxidation conditions of the aforementioned vegetable oil and comparison of antioxidant capacity of spices with the synthetic antioxidant. The present world is heading toward the concept of naturalism; this work could be helpful in replacing the synthetic antioxidants. It is important to note that several spices which are abundantly found in different areas of our country can have a better market with their growing utility, if found effective.

## 2 MATERIALS AND METHODOLOGY

Black pepper, clove, and ginger were bought from Dharan market, and oleoresins were extracted as per (Ranganna, 2007). Refined, bleached, and deodorized (RBD) sunflower oil was bought. Oleoresins of spices (200 ppm, based on extract weight) and TBHQ (200 ppm) were incorporated in the oil samples. The oil samples were then studied for the stability test under accelerated oxidation (70°C) test and heating at frying temperature (170°C).The heating test was performed for 90 min, and the samples were taken at 30, 60, and 90 min. The storage test was done for 72 hr, and the samples were taken at 4, 24, 48, and 72 hr. Blank oil was used as a control. Acid value (AV) and peroxide value (PV) of the samples were then analyzed. DDPH radical scavenging test was performed to find the antioxidant activity of the oleoresins and TBHQ.

### 2.1 Methodology

**Figure 1:**
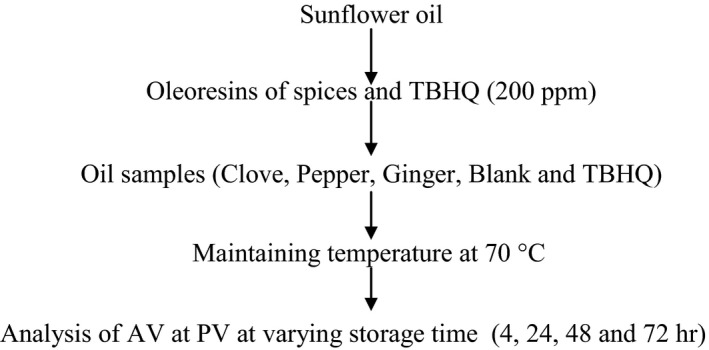
Flow diagram of effect of accelerated storage

**Figure 2:**
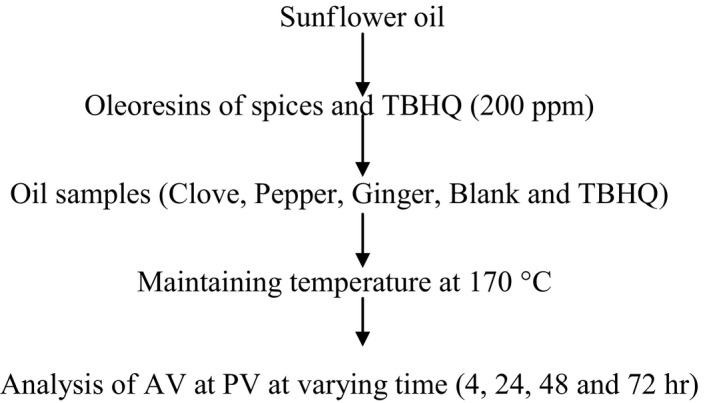
Flow diagram of effect of heating (at frying temperature 170°C) on AV and PV

The flowchart for methodology is shown in Figure 1 and Figure 2.

### 2.2 Analytical procedure

Estimation of peroxide value, acid value was done as per AOAC (2005). Estimation of cold test (winterization test) by AOCS (1967), Kries test, saponification value, free‐fatty acid was determined according to KC and Rai (2007).

### 2.3 DPPH radical scavenging test

Antioxidant Activity was measured by DPPH‐free radical scavenging method. The DPPH radical absorbs at 517 nm and the antioxidant activity can be determined by monitoring the decrease in this absorbance. The capacity of spices oleoresins and synthetic antioxidants to scavenge the lipid soluble DPPH radical is monitored at 517 nm (Singh, Singh, Saini, & Rao, 2008).

In this method, 1 ml methanolic solution of spice oleoresin at different concentration (5, 10, and 15 μl/ml) was mixed with 4 ml of 0.004% methanolic solution of DPPH. The absorbance was measured at 517 nm after 30 min. Control (without any additive) and standards (containing synthetic antioxidants viz. Butylated Hydroxyanisole (BHA), Butylated Hydroxytoluene (BHT), and Propyl Gallate (PG) in place of oleoresin) was also subjected to the same procedure for comparison. The capacity to scavenge the DPPH radical was calculated using the following equation:

$$\text{DPPH scavenging effect}\left(\%\right)\;=\;\left(A_{c}\;-\;A_{t}\right)/A_{c}\;\times \;100,$$

where *A*_c_ is the absorbance of control sample, and *A*_t_ is the absorbance of test sample.

Tert Butylhydroquinone (TBHQ) was used in making standard for comparison which is a modification from Singh et al. (2008).

Data were statistically processed by Genstat Discovery Edition 3, Genstat procedure library release PL15.2, version 7.22 DE (copyright 2008, VSN international Ltd.) for analysis of variance (ANOVA). Means of the data were separated (whether they are significant or not) using LSD (least square difference) method at 5% level of significance.

## 3 RESULTS AND DISCUSSIONS

### 3.1 Antioxidant activity

The antioxidant activities of the used antioxidants sources {(clove, black pepper, ginger) oleoresins, and TBHQ)} used were calculated by DPPH free radical scavenging method, at concentration of 5, 10, and 15 μl/ml. TBHQ showed the highest antioxidant activity (AA) in comparison to the other spices oleoresins.

**Figure 3:**
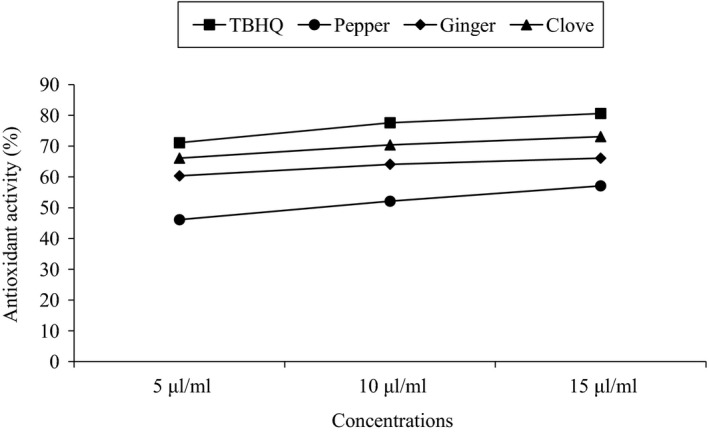
Antioxidant activities of all used antioxidants by DPPH‐free radical scavenging method

The order of AA was found to be, TBHQ > clove > ginger > black pepper. The values of AA were subjected to analysis of variance (ANOVA) for statistical analysis between individual samples at different concentration. Significant difference (*p* ≥ .05) was not found between them. ANOVA was also done between different samples at individual concentration. No significant difference was found for 10 and 15 μl/ml of concentration. Significant difference was found (*p* ≤ .05) at 5 μl/ml. The AA was found to increase steadily with increase in the concentration, as shown in Figure.3. As the concentration of the test solution was increased the value for antioxidant activity increases almost at the same pattern.

In a similar type of study performed by Sanjel (2010) the increasing pattern in antioxidant activity with increasing concentration of spices oleoresins and TBHQ was observed. The results were even similar to Wagensteen, Samuelsen, and Malterude (2004). Concentration‐dependent antioxidant activity was found in Zanthoxylum (Upadhyaya & Ashok, 2010); Black pepper and capsicum (Singh et al., 2008); TBHQ (Sanjel, 2010) in their study.

### 3.2 Physico‐chemical characteristics of RBD Sunflower oil

RBD sunflower oil was estimated for its important physico‐chemical property and the values so obtained are given in Table 1. According to AOCS (1967), specification for sunflower oil for the parameters, that is, saponification value must be in the range (188–194), peroxide value less than value 10 can be consumed, FFA value ≤ 0.25, Kries test ≤ 3.0. During cold test, the oil remained perfectly clear. From the analysis the used sunflower oil was of good quality, that is, was clear with no mass of crystal fats on the surface and was free of rancid. Saponification value was 188.3, peroxide value was 0.627, FFA was 0.082, acid value was 0.164, and Kries test 0.083 within the range.

**Table 1:** Physiochemical analysis of sunflower oil

Parameters	Sunflower oil^a^
Cold test (winterization test)	Good quality
Saponification value	188.3 (0.577)
Peroxide value (meqv/kg oil)	0.627 (0.0305)
% Free‐fatty acid as oleic acid	0.082 (0.0005)
Acid value (mg of KOH/gm of oil)	0.164 (0.001)
Kries test	0.083 (0.005)

a The values in the Table 1 are the means of triplicates. Figures in the parentheses are the standard deviation.

### 3.3 Effect of heating (at frying temperature, 170°C) on AV of different samples

**Figure 4:**
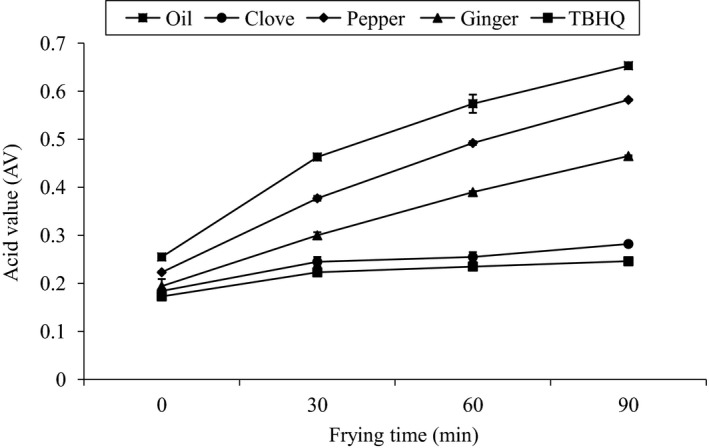
Effect of heating (at frying temperature 170°C) on AV of different samples

Acid value (AV) was found to be increasing with the increase in heating time for all samples except for TBHQ at 30 and 60 min. Control exhibited the highest AV at 0, 30, 60, and 90 min followed by pepper, ginger, clove, and TBHQ, respectively. At the final stage of heating (90 min), the AV for blank, clove, pepper, ginger, and TBHQ were 0.65 ± 0.0026, 0.28 ± 0.002, 0.58 ± 0.0015, 0.46 ± 0.001, and 0.25 ± 0.001, respectively, maximum value was found for blank and least for TBHQ as shown in Figure 4.

ANOVA was done between AV of individual sample with varying heating time (0, 30, 60, and 90 min). No significant difference (*p* ≥ .05) was found between individual samples at different intervals of times, that is, 0, 30, 60. and 90 min. Again, ANOVA was done between AV of each sample at a particular time. Significant difference (*p* ≥ .05) was found between individual samples except clove and TBHQ showed no significant difference (*p* ≥ .05) among them at each time, except at 90 min.

On a similar type of experiment done in Palm oil by Sanjel (2010), there was sharp rise in AV at initial stages but with the increasing time it slowed down. Similar results were obtained by Singh, Maurya, and Catalan (2005), Liu, Han, Lee, Hsu, and Hou (2003) in the study with different types of oil.

### 3.4 Effect of heating (at frying temperature 170°C) on PV of different samples

**Figure 5:**
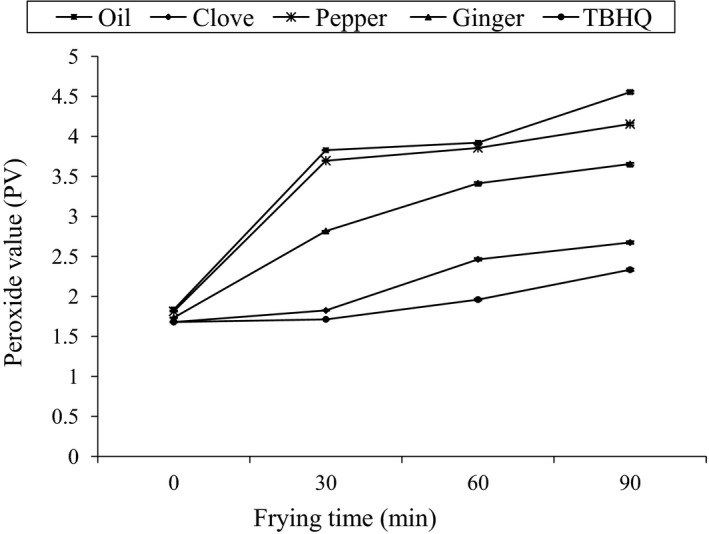
Effect of heating at frying temperature (170°C) on PV of different samples

Peroxide value (PV) was found to be increasing with the increase in heating time for all samples, higher PV was observed for control followed by pepper, ginger, clove, and TBHQ, respectively, but at 0 min TBHQ had higher PV than clove. A regular increase in PV as a function of storage time was observed for all the samples at all intervals, shown in Figure 5. At the final stage of heating (90 min), PV was in the range between 4.55 ± 0.0015, 2.67 ± 0.0015, 4.15 ± 0.0015, 3.65 ± 0.0015, and 2.33 ± 0.0015 for blank, clove, pepper, ginger, and TBHQ, respectively; maximum value was found for blank and least for TBHQ as shown in Figure 5. The result was similar to Sanjel (2010); had conducted his study in palm olein oil with incorporation of different spices oleoresins and TBHQ with frying potato chips in the oil samples.

ANOVA was done between PV of individual sample with varying heating time (0, 30, 60, and 90 min). No significant difference (*p* ≥ .05) was found between blank (oil only) and pepper at different intervals of times, that is, 0, 30, and 60. Again, ANOVA was done between PV of each sample at a particular time period (30; 60; 90 min) of heating periods. Significant difference was found (*p* ≥ .05) in PV in each sample at particular time, except at 30 and 60 min for blank and pepper.

### 3.5 Effect of accelerated oxidation storage (70°C) on AV of different samples

**Figure 6:**
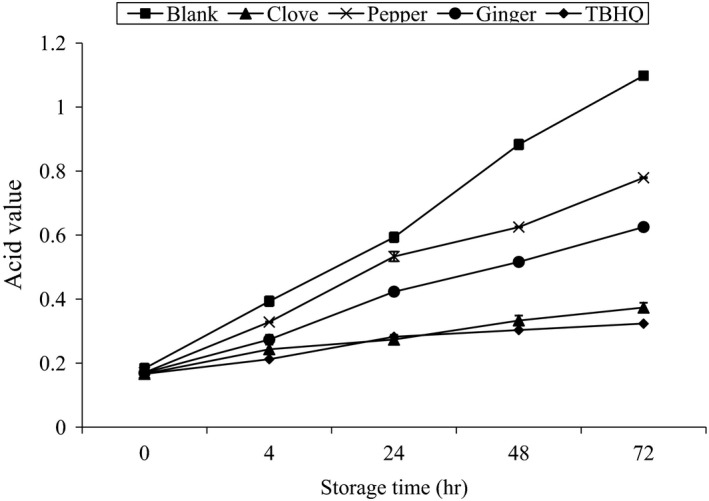
Effect of accelerated oxidation (70°C) storage on AV of different samples

Acid Value (AV) went on increasing with the increase in storage period for all the samples. Control exhibited the highest AV at all the storage period. At storage period of 4, 48, and 72 hr control exhibited highest AV followed by pepper, ginger, clove, and TBHQ, respectively, but at 24 hr of storage TBHQ have higher AV than clove. Clove and TBHQ have similar result at 0 min of storage. At the final storage time (72 hr) the AV for blank, clove, pepper, ginger, and TBHQ were 1.098 ± 0.0015, 0.373 ± 0.0015, 0.779 ± 0.0015, 0.625 ± 0.0015, and 0.323 ± 0.0015, respectively. The pattern of AV rise is as shown in Figure 6.

In blank and clove there was significant difference (*p* ≤ .05) between AV at different time periods but there was no significant difference in clove and TBHQ. ANOVA was done between AV of different samples at different time intervals, that is, 4; 24; 48; 72 hr. Significant difference (*p* ≤ .05) was found between AV of different samples, except at 0, 4, and 24 hr for clove and TBHQ. The changes in AV pattern are similar with the result obtained by (Iqbal & Bhanger, 2007). Iqbal and Bhanger (2007) had used sunflower oil and garlic extract was used in stabilizing the oil at accelerated oxidation condition, the pattern of AV change was quite irregular as of my result and the rise of AV in blank was in increasing trend.

### 3.6 Effect of accelerated oxidation (70°C) storage on PV of different samples

**Figure 7:**
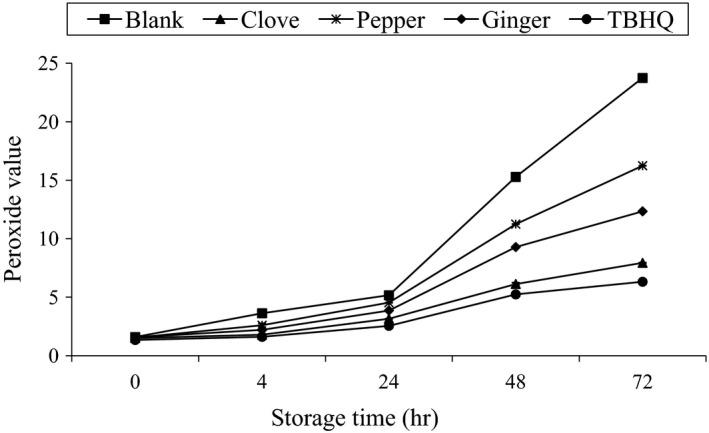
Effect of accelerated oxidation (70°C) storage on PV of different samples

Peroxide value (PV) went on increasing with the increase in storage period for all the samples. At all stages of storage time, higher PV was observed for control followed by pepper, ginger, clove, and TBHQ, respectively, except at 0 and 4 hr of storage after control higher PV was observed of ginger, pepper, clove, and TBHQ, respectively. At the final storage time (72 hr) the PV for blank, clove, pepper, ginger, and TBHQ were 23.74 ± 0.0015, 7.93 ± 0.0015, 16.2 ± 0.006, 12.34 ± 0.0096, and 6.32 ± 0.0015, respectively; maximum value was found in the control while least in TBHQ as shown in Figure 7.

ANOVA was done between PV of each sample at a particular time. Significant difference was not found (*p* ≥ .05) at individual samples at different interval of storage period except at 48 and 72 hr. Again, by ANOVA, the changes in PV pattern with accelerated oxidation time for each stabilized samples and blank was observed. At 48 hr of storage blank and pepper were out of range of specification (PV ≤ 10 m eqv/kg oil). At 72 hr of storage blank, pepper and ginger showed unacceptable range of specification for sunflower oil. For all samples above 4 hr of storage time, there was significant rise (*p* ≤ .05) in PV up to the analyzed time of 72 hr. There was an increasing trend of PV rise up to 48 hr, but this rate slightly decreased for all samples from storage time of 48 hr to 72 hr except blank which is similar to the observation of Mohdalay, Sarhan, Smetanska, and Mahmoud (2010). The slower rate of increase in PV with the increasing time may be due to volatilization of some breakdown products of lipid hydroperoxides, formed in the primary stages of oxidation.

## 4 CONCLUSIONS

The natural antioxidants from plants have been solitary studied for their antioxidant potential in the vegetable oil replacing synthetic antioxidants. The antioxidant activity was found to be in the order TBHQ > clove > ginger > pepper, by DPPH radical scavenging activity Method. The effectiveness of the used oleoresins and TBHQ to control AV and PV was found in the order TBHQ > clove > ginger > pepper. A direct correlation was found between antioxidant activity and TBHQ in controlling AV and PV of the sunflower oil. Clove was found to be the most effective to stabilize sunflower oil among the used spices oleoresins with respect to TBHQ.

## CONFLICT OF INTEREST

None declared.
